# Needle tip insertion technique for accurate and safe puncture in endoscopic ultrasound-guided tissue acquisition

**DOI:** 10.1055/a-2477-2789

**Published:** 2024-12-12

**Authors:** Ryota Sagami, Yasuhisa Hiroshima, Yoshifumi Azuma, Hiroaki Tsuji, Hidefumi Nishikiori, Kazuhiro Mizukami, Kazunari Murakami

**Affiliations:** 1 12995 Gastroenterology, Faculty of Medicine, Oita University, Oita, Japan; 2 157533 Gastroenterology, Oita San-ai Medical Center, Oita, Japan

Endoscopic ultrasound-guided tissue acquisition (EUS-TA) is the gold standard for diagnosing pancreatic and gastrointestinal submucosal tumors 1 . However, inaccurate puncture can occasionally lead to adverse events or insufficient tissue samples 2 3 4 . In standard EUS-TA procedures, puncture is performed to confirm the alignment of the external sheath with the target lesion; however, several technical challenges can arise ( Fig. 1 **a** ). Although the needle direction and distance to the target lesion are typically controlled by focusing on the external sheath, complications such as needle deviation from the expected direction, gastrointestinal membrane mobility, and breathing-induced target movement can lead to unsuccessful or inadequate punctures ( Fig. 1 **a** , Fig. 2 **a, b** ). Misalignment of the endoscope or external sheath can also result in needle loss from the EUS view ( Fig. 2 **c, d** , Video 1 ). Unintended puncture routes may subsequently increase the risk of adverse events, including vascular injury.

**Fig. 1:**
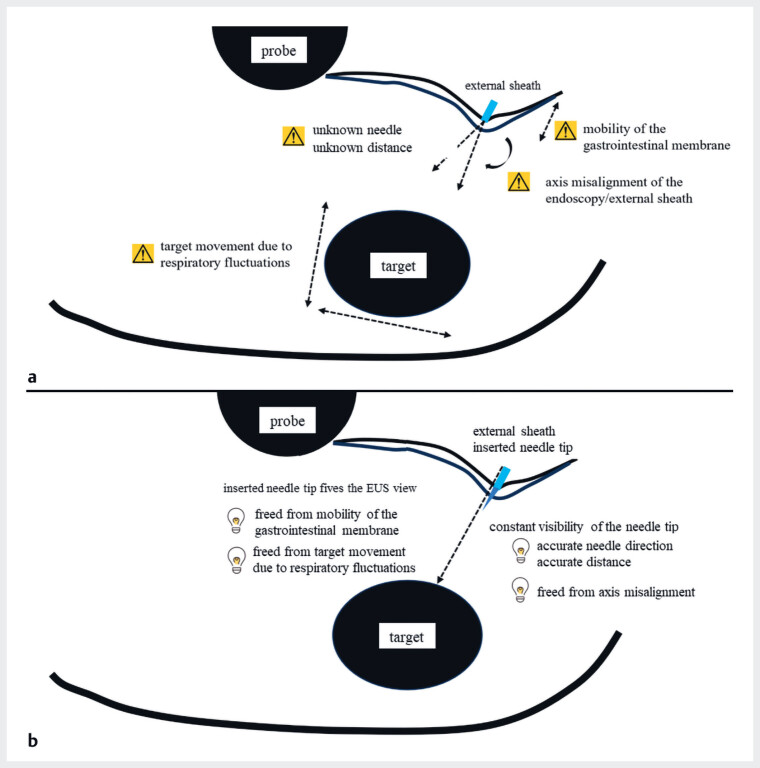
Endoscopic ultrasound-guided tissue acquisition (EUS-TA) and needle tip insertion method. **a** Standard EUS-TA technique. Puncture is attempted to confirm the positions of the external sheath and the target lesion. The needle direction and distance to the target lesion are determined based on the external sheath position. However, unsuccessful or inadequate punctures can occur due to needle deviation from the intended direction or distance, gastrointestinal membrane mobility, breathing-induced target movement, and endoscope or sheath misalignment, which can cause the needle to exit the EUS view. **b** Needle tip insertion method: The needle tip is inserted a short distance from the external sheath, and the inserted needle tip crimps the gastrointestinal membrane. The needle tip insertion method provides a stable puncture view that is unaffected by respiratory fluctuations or mucosal mobility, enabling accurate measurement of the target distance. Continuous visibility of the needle tip allows precise measurement of distance and assessment of the needle puncture direction and route, even when endoscope or external sheath misalignment occurs.

**Fig. 2:**
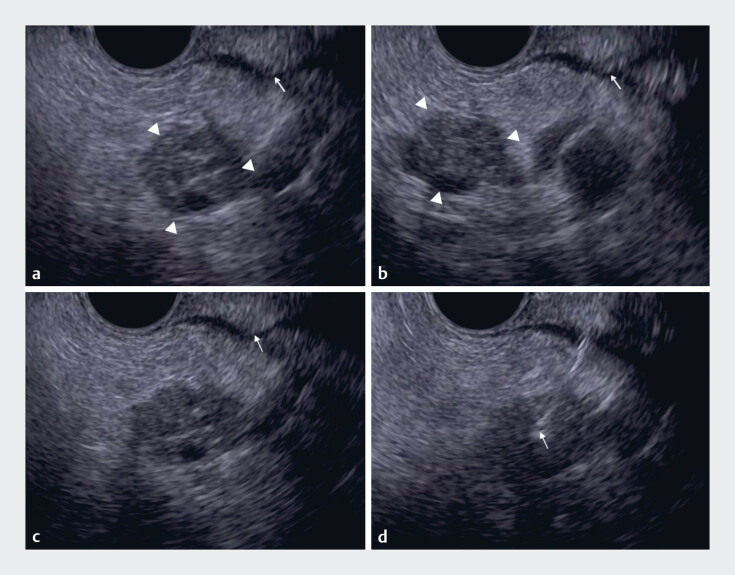
Needle loss from the endoscopic ultrasound (EUS) view due to axis misalignment of the endoscopy or external sheath. **a–b** The external sheath tip (arrow) and the target tumor (arrowheads) are visible ( **a** ) and demonstrated significant movement within the EUS view ( **b** ). **c** At the time of puncture, although the external sheath tip is visible, the needle itself is not visible. **d** Misalignment between the endoscope and needle axes is evident in the EUS view. By adjusting the axis through slight twisting of the endoscope, the needle becomes visible.

Endoscopic ultrasound-guided tissue acquisition (EUS-TA) using the needle tip insertion method (NTIM) enables accurate puncture with continuous needle tip visibility, unaffected by respiratory fluctuations, mucosal or target mobility, or axis misalignment.Video 1

The needle tip insertion method (NTIM) is recommended to prevent such punctures ( Fig. 1 **b** ). In NTIM, the needle tip is inserted a short distance from the external sheath and crimped to the gastrointestinal membrane ( Fig. 3 **a** ). Needle crimping provides a stable puncture view unaffected by respiratory fluctuations and enables accurate measurement of the target distance, independent of mucosal mobility ( Fig. 3 **b** ). Continuous visibility of the needle tip facilitates more precise distance measurements and allows for accurate assessment of the needle’s puncture direction and route, even when endoscope or external sheath misalignment occurs ( Fig. 3 **c** , Video 1 ).

**Fig. 3:**
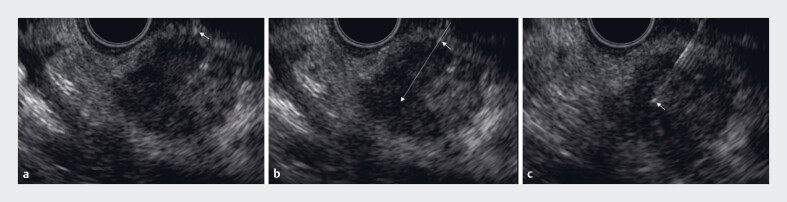
Needle tip insertion method for a large tumor in the pancreatic body. **a** The external sheath tip (arrow) is visible, but the needle direction and distance between the sheath and tumor cannot be accurately determined. **b** The needle tip is inserted a short distance from the external sheath (short arrow), and the gastric membrane and tumor are fixed. In addition, the needle direction can be anticipated (long, dotted arrow). **c** The needle and its tip (arrow) remain continuously visible during puncture and back-and-forth movements.

NTIM is particularly beneficial for accessing technically challenging pancreatic tumors, such as those located deep within the pancreas or far from the puncture site ( Fig. 4 **a, b** ). It also maintains stable visibility when targeting highly mobile microtumors in the pancreatic tail ( Fig. 4 **c, d** , Video 1 ). Additionally, NTIM is useful for puncturing submucosal and small hepatic tumors ( Fig. 5 , Video 1 ). Therefore, incorporating NTIM into EUS-TA procedures may enhance both accuracy and safety.

**Fig. 4:**
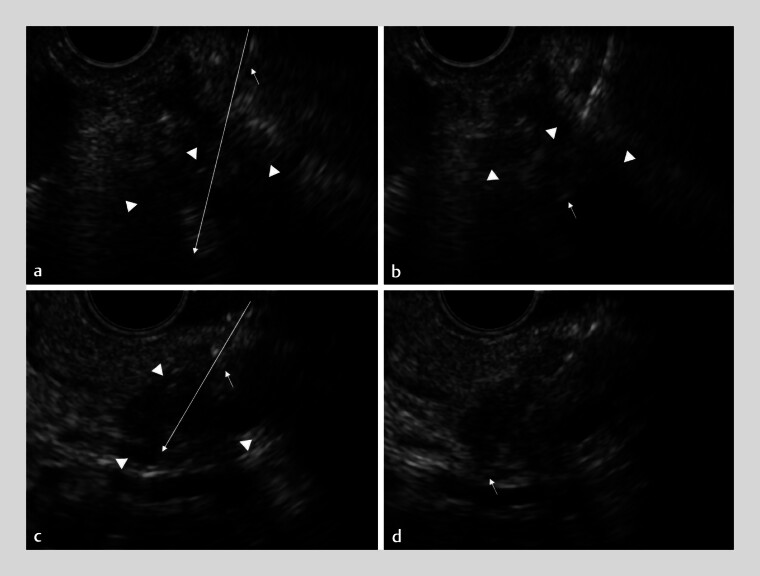
Needle insertion method is useful for puncturing technically challenging pancreatic tumors, such as those located deep in the pancreas far from the needle and highly mobile microtumors in the pancreatic tail with stable visibility. **a** The target low echoic area (arrowheads) is distant from the puncture site; however, the needle direction can be predicted (long, dotted arrow). The needle tip (short arrow) remains continuously visible during puncture and back-and-forth movements. **b** A microtumor in the pancreatic tail (arrowheads), tightly surrounded by vessels and showing significant movement in the endoscopic ultrasound view, can be accurately punctured with a predictable needle trajectory (arrow). **c** The target tumor (arrowheads), needle tip (short arrow), and predicted trajectory of the needle (long, dotted arrow) can be observed. **d** The needle (arrow) is seen puncturing the target tumor.

**Fig. 5:**
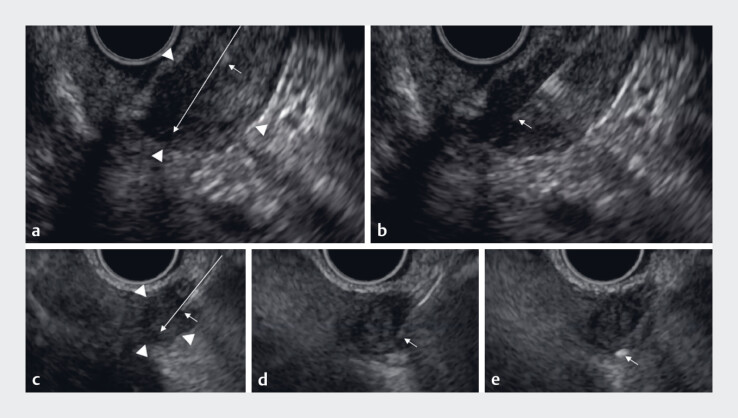
Accurate puncture of submucosal tumor and small liver tumors. **a** The mobile submucosal tumor (arrowheads) can be accurately punctured with a predictable trajectory (long, dotted arrow) of the needle (short arrow). **b** Needle tip (arrow) punctured the submucosal tumor. **c** Liver microtumor (arrowheads) can be accurately punctured with the predicted trajectory (long, dotted arrow) of the needle (short arrow). **d–e** The needle (arrow) is inadequately inserted into the tumor ( **d** ); however, the needle tip remains visible ( **e** ), and the needle can be inserted more deeply after confirmation of the location of the needle tip.

Endoscopy_UCTN_Code_TTT_1AS_2AF

## Notes

Endoscopy E-Videos https://eref.thieme.de/e-videos
*E-Videos* is an open access online section of the journal *Endoscopy* , reporting on interesting cases and new techniques in gastroenterological endoscopy. All papers include a high-quality video and are published with a Creative Commons CC-BY license. Endoscopy E-Videos qualify for HINARI discounts and waivers and eligibility is automatically checked during the submission process. We grant 100% waivers to articles whose corresponding authors are based in Group A countries and 50% waivers to those who are based in Group B countries as classified by Research4Life (see: https://www.research4life.org/access/eligibility/ ). This section has its own submission website at https://mc.manuscriptcentral.com/e-videos .
